# Experience with a triplex arbovirus nucleic acid test (NAT) at a Canadian Public Health Laboratory

**DOI:** 10.1186/s12879-021-06842-w

**Published:** 2021-11-10

**Authors:** Saugata Choudhury, Raymond Tellier, Kevin Fonseca, Byron M. Berenger

**Affiliations:** 1 Dept of Pathology and Laboratory Medicine, Cumming School of Medicine, University of Calgary, Calgary, AB Canada; 2 Public Health Laboratory, Alberta Precision Laboratories, Calgary, AB Canada; 3 Dorevitch Laboratories, 18 Banksia Street, Heidelberg, Melbourne, Australia; 4 Div of Infectious Diseases, Dept of Medicine, McGill University Montreal, Montreal, Canada; 5 Dept of Microbiology, Immunology and Infectious Diseases, Cumming School of Medicine, University of Calgary, Calgary, AB Canada

**Keywords:** Arbovirus, NAT, Dengue, Zika

## Abstract

**Background:**

Dengue, chikungunya and zika infections occur in tropical and subtropical regions of the world. We describe the utilization of an in-house nucleic acid test (NAT) targeting all three viruses for febrile returning travelers in Alberta, Canada.

**Methods:**

NAT was performed until 40 days from symptom onset or exposure due to the prolonged duration of zika virus RNA detection. From Sept 1, 2017 to August 31, 2019, 2552 specimens from 1932 patients were tested.

**Results:**

Approximately 2% of patients tested were NAT positive for dengue virus (n = 42), chikungunya virus (n = 4), and zika virus (n = 1). The majority presented with fever, myalgia and rash. Regions with the most frequent travel included SouthEast Asia (68.5%), South America (25%) and the Caribbean (6.5%). Ct values were stronger (~ 1.5 logs) for patients within 1–3 days following onset of clinical symptoms than those presenting later. Nineteen patients had urine and plasma submitted; 5 were positive for both specimens and 2 were positive only for dengue virus in the urine. Also, Ct values were lower for plasma when compared to the corresponding urine. RNA was detected until 10 days and 5 days post-exposure in plasma and urine respectively for dengue virus.

**Conclusions:**

Owing to dengue viremia detected beyond the conventional 7 days and low levels of circulating zika virus globally, a cutoff of 14 days from symptom onset to NAT is sufficient to diagnose acute cases. Inclusion of a zoonotic history form that collects appropriate clinical history results in improved test utilization.

## Background

Arboviruses are a taxonomically diverse group of viruses transmitted by arthropod vectors. Of these, dengue virus (DENV), chikungunya virus (CHIKV) and zika virus (ZIKV) are considered the most epidemiologically important arboviruses globally [1].

Recently, these mosquito-borne arboviruses have been reported in areas different from their endemic regions. DENV is considered largely a tropical disease. However, recently outbreaks have been described across the US in Texas, Florida and Louisiana [2]. ZIKV was responsible for a large epidemic in French Polynesia in 2013; reaching the American continent in the same year [3] before spreading to 87 countries and territories throughout the world by July 2019 [4]. Likewise, CHIKV was quiescent for about 30 years before it re-emerged in 2005–2006 as a massive epidemic in the Indian Ocean islands [5]. It spread subsequently to the Americas in 2013 [6].

These three arboviruses share common vectors namely *Aedes aegypti* and *A. albopictus*. They can thus co-circulate as evidenced in the recent outbreak in the Americas [7]. They are also characterised by overlapping signs and symptoms including fever, maculopapular rash and headache [8].

While infections caused by either of these three viruses, can be asymptomatic, they may also have adverse effects including neurological disorders [9]. Cases of microcephaly have also been documented following maternal asymptomatic infection with ZIKV [10] contributing to the overall picture of Congenital Zika Syndrome [11].

Factors like urbanization, globalization, and global warming have facilitated the expansion of the *A. aegypti* and *A. albopictus* vectors from their areas of endemicity to other regions [12, 13]. This greatly enhances the risk for arboviral infections in regions where the local population is immunologically naïve. Moreover, the global population is at peril from different modes of transmission including sexual [14], perinatal [15] and transfusion [16], especially since no vaccine against ZIKV or CHIKV currently exists.

Infection by all three of these viruses can be detected using acute and convalescent antibody responses, but this can be problematic due to cross-reactivity of antibodies within the *Flaviviridae* family (especially for ZIKV and DENV) and in cases of re-infection with a heterotypic DENV. Nucleic acid testing or antigen-based testing are a more accurate method to diagnose acute infection, but the duration of viremia from symptom onset is short for DENV (on average 1–3 days, max 7 days) [17] and CHIKV (8 days) [18]. In ZIKV infection however, viremia can be detected up to 41 days [19]. Overlapping symptomology, but different duration of viremia poses a unique diagnostic concern because where all three viruses are present they all need to be tested for at the same time.

In 2016, the Alberta ProvLab implemented a triplex Real time Nucleic Acid Test (NAT) [20] for ZIKV, DENV 1–4, and CHIKV. These arboviruses have overlapping symptomology and geographical distribution (especially after the 2016 ZIKV outbreak). The 95% LOD by this triplex NAT is 15 copies/reaction for DENV-1 and less than 10 copies/reaction for all the rest of the viruses. This assay is adaptable to a variety of specimen types including serum, plasma, urine, placental tissue, brain tissue and amniotic fluid.

In response to the ZIKV epidemic in the Americas and as Albertans tend to travel widely especially to endemic areas in Asia and due to the overlapping symptomology of these three viruses, we implemented standard criteria on when to perform a triplex NAT for ZIKV, DENV, and CHIKV. The criteria for performing the NAT for the period Sep1, 2017–Sep 30, 2018 was until 14 days from symptom onset or exposure. The cut-off for NAT was extended to 40 days for the period Oct 1, 2018–Aug 31, 2019. This study describes the experience of the Alberta Public Health Laboratory (ProvLab) in molecular testing for ZIKV, DENV, and CHIKV using a standardized case history form during 2017–2019 following the height of the ZIKV epidemic.

The primary purpose of this study was to improve utilization of arbovirus NAT. This was achieved by determining the positivity rate and if the expanded algorithm could detect more cases of and assess the relative viral loads at different time points from symptom onset/exposure or in different sample types. We also describe the travel history, symptoms and relative viral loads in different sample types or time of diagnosis from symptom onset/exposure, and the serological results of positive cases.

## Methods

### Criteria for testing and patient population

Clinicians ordering arbovirus testing (except West Nile Virus serology/NAT during the local season i.e. 1st June–31st Dec) must submit a zoonotic history form (in the Electronic Medical Record or on paper) or provide equivalent information on the requisition. A Canadian College of Clinical Microbiology certified microbiologist or Royal College of Canada certified Medical Microbiologist reviewed the history forms for demographic data, symptoms, pregnancy status, travel destination and date of return to Canada relative to symptom onset to determine the appropriate testing (i.e. serology and/or NAT). In 2016, the Public Health Agency of Canada published guidelines for ZIKV testing in pregnant females [21]. A serological test and/or NAT was recommended depending on presence of symptoms and/or signs suggestive of infection and date from onset of exposure.

In our study, both asymptomatic pregnant patients as well as patients with symptoms and/or signs suggestive of infection (regardless of pregnancy status) were tested.

Those with symptoms that were not consistent with arbovirus infection or who had no recent travel to an area with known endemicity or risk of arbovirus transmission were not tested, irrespective of pregnancy status or the desire to conceive.

From Sept 1, 2017 to Sep 30, 2018 ZIKV, DENV and CHIKV NAT was done if travel was within 14 days of symptom onset or date of return as per recommendations by Committee to Advise on Tropical Medicine and Travel [22]. Oct 1, 2018 and onwards, the NAT was done if the date of return was 40 days within sample collection. This was done in response to the study by Paz-Bailey et al. [19]. Urine was requested for arbovirus NAT due to findings of prolonged shedding of ZIKV in urine [23]. ProvLab provides arbovirus testing for the entire province of Alberta (population approximately 4 million).

### Nucleic acid testing

Viral RNA from samples were extracted using the easyMAG^®^ automated extractor (BioMerieux, Quebec, Canada), according to manufacturer’s instructions. The input volumes for plasma and cerebrospinal fluid were 200 µl and the output volume was 55 µl. For urine the corresponding volumes were 200 µl and 110 µl respectively. A one-step RT-PCR was then performed employing TaqMan^®^Fast VirusOne-Step RT-PCR Master Mix (ABI) according to the protocol of Pabbaraju et al*.* [20].

### Serology testing for ZIKV, DENV, and CHIKV

Sera was tested for Dengue IgM and IgG antibodies using ELISA (DENV IgM capture DxSelect and DENV IgG DxSelect, Focus Diagnostics, Cypress, CA). CHIKV antibodies were tested employing the Euroimmun IgM/IgG (Lubeck, Germany) assays. Meanwhile, Zika IgM was assayed using either an in-house CDC MAC ELISA [24] or the Zika Detect™ 2.0 IgM Capture ELISA (InBios International Inc., Seattle, WA, USA). Euroimmun ELISA (IgG) kits were employed for the Zika IgG.

### Data extraction and review

Information recorded in the laboratory information system on all arbovirus NAT orders and the original requisitions/history forms were reviewed. This was part of a quality improvement exercise to determine the efficacy of the time period for NAT testing from 40 days since symptom onset/exposure. The study protocol was approved by the institutional review board and ethics committee of the University of Calgary (Approval number: REB 17-0662).

### Statistical analysis

All statistical analyses were performed using R version 3.5.1 [25]. The packages used were ggplot2 and ggpubr.

## Results

A total of 2552 specimens from 1932 patients were tested by the arbovirus NAT during the study period. Simultaneously, 5130 NAT requests were deemed to be unnecessary following a review of their zoonotic history forms and were thus cancelled. 61.65% (n = 1191) of the total NAT tests performed were for females. The specimen types tested were predominantly plasma (78.2%, n = 1996) and urine (19.1%, n = 489). Plasma and urine were submitted simultaneously for 431 patients. Other specimen types included tissue (n = 49), cerebrospinal fluid (n = 16) and other body fluids (n = 2).

Forty-seven patients (2.4%) were positive by the NAT (Table 1). The overwhelming majority of positives were due to DENV (n = 42, 89.3%). Four patients were positive for CHIKV and only one for ZIKV. Nineteen of the 47 NAT positive patients had both plasma and urine submitted*.* Of these 19 patients, 12 were positive by the NAT only in the plasma while the corresponding urine was negative. Five patients were positive both in the plasma and urine. In 2 patients, only the urine was positive for DENV. A negative result was obtained on the NAT for the remainder of the patients (n = 412/431) who had a paired plasma/urine submitted.

**Table 1:** Number of patients positive for each target on the arbovirus NAT stratified by specimen type

Agent (n)^a^	Plasma only	Urine only	Plasma and urine
Dengue (42)	37	2	3
Chikungunya (4)	3	–	1
Zika (1)	–	–	1

^a^n = number of patients

**Fig. 1:**
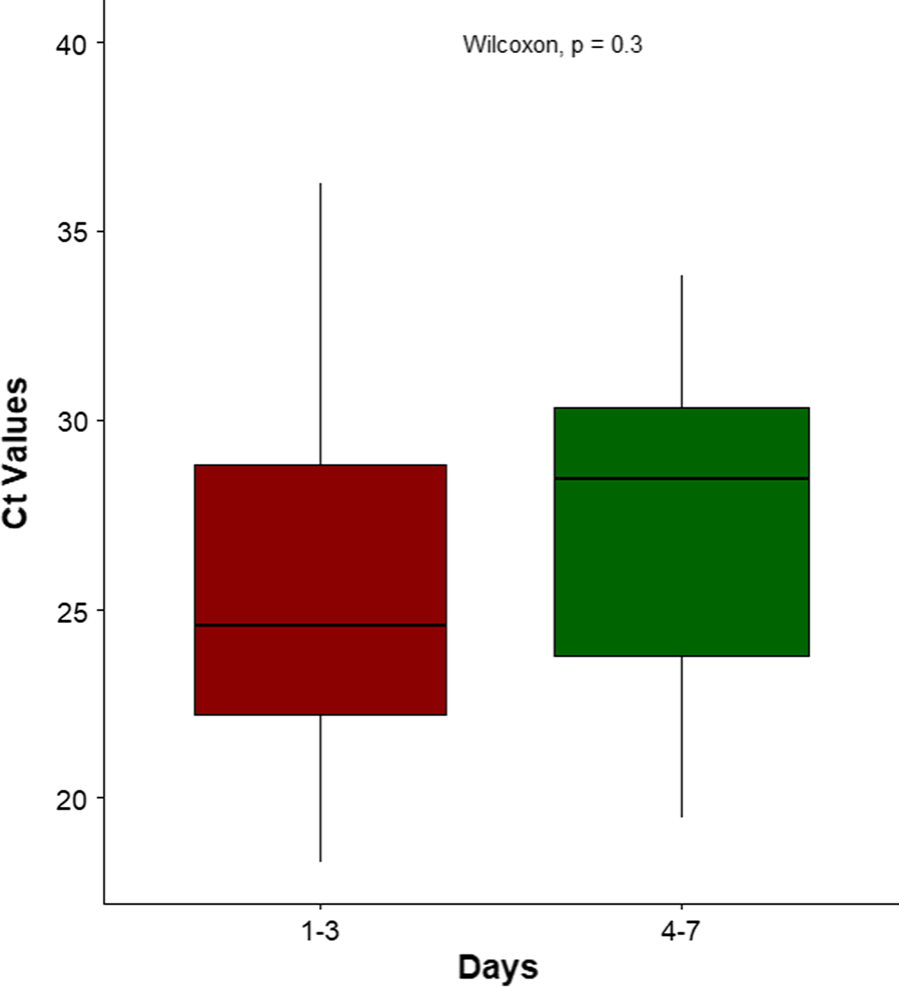
Ct values for dengue on the NAT for plasma stratified by day of testing following symptom onset. *Ct values for patients presenting on the 8th and 10th day following symptom onset were 30.22 and 25.34 respectively (within each box, horizontal black lines denote median values; boxes extend from the 25th to the 75th percentile of each group’s distribution of values)

In patients wherein both the specimen types were positive by NAT (n = 5) (Table 2), the Cycle threshold (Ct) values were consistently lower for the plasma when compared to the corresponding urine. The median Ct value for DENV was 24.58 (interquartile range 17.92–31.23) for patients tested between 1 and 3 days after symptom onset (Fig. 1) while those tested at 4–7 days from symptom onset had a median Ct value of 28.45 (interquartile range, 21.91–34.98). Although the Ct values do reflect a difference exceeding the total allowable error, the difference was not statistically significant (Wilcoxon ranked-sum test, p = 0.3) because of the low n value.

**Table 2:** Intrapatient comparison of cycle threshold (Ct) values for plasma and urine for each target on the arbovirus NAT

Agent	Patient	Plasma	Urine
Dengue	1	20.33	38.49
	2	24.44	38.93
	3	33.52	37.8
Chikungunya	1	17.74	33.66
Zika	1	34.92	36.24

We detected RNA up to a maximum of 10 days post-symptom onset in the plasma for DENV NAT positive patients (median = 5 days, 10th percentile = 1 day and 90th percentile = 7 days). The maximum duration of detection in urine was 5 days post-symptom onset across all targets (n = 2).

Dengue serology results were available for 36 of the 42 NAT cases. Sixteen of these had a serological profile not consistent with an acute infection, underscoring the role of the NAT in definitive diagnosis. Seven specimens were Dengue IgM negative and IgG positive which was consistent with a picture of remote infection while 9 had both IgM/IgG negative.

Fever (n = 26) and myalgia/arthralgia (n = 15) were the predominant symptoms in patients with NAT confirmed DENV infection. Rash (n = 11) was also a common presenting feature in confirmed cases. Gastrointestinal symptoms were relatively uncommon (n = 4).

Not surprisingly, due to the association with congenital abnormalities, overall more specimens were received for ZIKV testing from females than from males. Twenty percent (n = 246) of women tested for arboviruses by NAT were pregnant women tested for ZIKV and none had confirmed infection.

The majority of the patients (68.5%, n = 32) with a positive NAT had travelled to South-East Asia. The next common destination was South America (25%, n = 12). Sporadic infections were diagnosed in returning travelers to the Caribbean (6.5%, n = 3). All these infections served as sentinel indicators of ongoing low-level transmission in these geographic regions.

## Discussion

We hypothesized that adopting the change from the findings by Paz-Bailey et al. [19] to our cut-off post symptom onset for the arboviral NAT might diagnose more cases. This was not found to be the case, which was most likely because the algorithm was implemented as the ZIKV epidemic in the Americas was declining [26]. However, we demonstrate that there is merit in performing NAT for DENV beyond 1 week of symptom onset. This is particularly crucial since it has been shown that defervescing patients with dengue viral loads as low as 10^4^ copies/ml in their blood are infective to the *Aedes* vector [27].

Dengue and chikungunya viremia are reported to last 7–8 days [28, 29]. Our cohort of positive NATs had patients until 10 days from symptom onset. Therefore, performing DENV NAT more than a week from symptom onset can diagnose more cases. Changing from a cutoff of 14 days from symptom onset to 40 days appeared to have no impact on our ability to detect DENV, CHIKV or ZIKV infection. This is attributable to the low number of ZIKV cases during the study period globally. DENV infections vastly outnumbered those due to ZIKV on the arbovirus NAT. This is not surprising in light of the recent decrease in the number of cases of ZIKV [30]. ZIKV cases in the Americas have declined by at least 30-fold from its zenith in 2016 and the ratio of DENV to ZIKV cases is currently reported to be 200:1 [31]. Before the study period from 2016 to 2017 and when 14 days or less were used as a guide for NAT testing, we had 50 cases of ZIKV infection [32]. Expansion to the 40-day window may be worth exploring if there is a resurgence of ZIKV, but our data and current knowledge on dengue and chikungunya viremia indicate that 14 days is suitable.

The IgM antibody response in ZIKV infection is longstanding; a recent study detected Zika IgM 25 weeks post infection [33]. This is compounded by cross reactivity across the *Flaviviridae* family [34]. A positive serology has to thus undergo confirmation by the labor-intensive Plaque Reduction Neutralization Test (PRNT) (performed at the Zoonotic Division, National Microbiology Laboratory, Winnipeg) which has a turnaround time of 21 days. Although neutralizing antibodies are deemed to be specific, in patients with prior flavivirus infections a rise in neutralizing antibodies may be elicited through the “original antigenic sin” phenomenon (Hoskins effect). The lengthy timeframes and relative uncertainty linked to serological modalities pose a particular challenge for pregnant women who require intervention and monitoring as soon as possible given its putative association with birth defects (Congenital Zika Syndrome). Thus, a rapid negative result on the NAT by the extended cut-off might prove reassuring to both the patient and the attending physician. A corresponding negative result on serology would be of considerable value for confirmation.

Furthermore, for the other targets on this triplex assay, namely CHIKV and DENV which are characterized with a relatively brief duration of viremia, an extended cut- off would potentially facilitate the detection of more acute cases even with low levels of viremia by the NAT. A positive NAT, albeit weak, in conjunction with corroborative serology might clinch a diagnosis for the rare patient who presents in the later stages of the illness. One cannot possibly undermine the epidemiological and public health implications of such a finding, though some may argue about its relevance beyond a clinically relevant timeframe.

## Conclusion

Owing to the lowered pre-test probability for ZIKV, we conclude that the yield from this extended cut-off was negligible when compared with the laboratory resources expended. The very low number of positives for ZIKV on the NAT argues against extended screening. Our observation supports the latest guidance from the CDC [31] which advocates for decreased testing for ZIKV in this post-epidemic scenario. The zoonotic history form employed here proved to be a powerful tool in deciding upon the type of testing (serology v/s molecular) depending on variables like the travel history and timeline of exposure. The zoonotic form also served as an effective screening aid given that a majority of the NAT requests received were deemed unnecessary.

Last but not least, it has been amply demonstrated, that for the flaviruses [23], urine offers a number of advantages over plasma, because of ease of collection and prolonged shedding reported for ZIKV. Urine as a specimen type, though, was particularly helpful for diagnosing two individuals with dengue virus infection when the corresponding plasma was negative.

## Data Availability

The datasets used and/or analysed during the current study are de-identified and available from the corresponding author on reasonable request.

## Abbreviations

CDC: Centre for Disease Control and Prevention
CHIKV: Chikungunya virus
Ct: Cycle threshold
DENV: Dengue virus
ELISA: Enzyme Linked Immuno Sorbent assay
NAT: Nucleic acid test
PRNT: Plaque Reduction Neutralization Test
RNA: Ribonucleic acid
RT-PCR: Reverse-Transcriptase Polymerase Chain Reaction
ZIKV: Zika virus
